# Many Moving Parts: New Transplant Allocation Models Are Associated With Increased Organ Travel and Potential Climate Implications

**DOI:** 10.1111/ctr.70426

**Published:** 2025-12-22

**Authors:** Kevin Gianaris, Arrey‐Takor Paul Ayuk‐Arrey, Jonathan A. Fridell, Katherine Ross‐Driscoll

**Affiliations:** ^1^ Indiana University School of Medicine Indianapolis Indiana USA; ^2^ Center For Health Services Research Regenstrief Institute Indianapolis Indiana USA; ^3^ Indiana University School of Medicine Department of Surgery Indianapolis Indiana USA

**Keywords:** allocation, climate, emissions, environmental impact, health policy, organ transplantation, public policy, transportation, UNOS

## Abstract

**Background:**

Organ allocation has recently changed to acuity circle (AC)‐based policies. This study aimed to quantify changes in travel distance and potential environmental impacts of AC policies.

**Methods:**

Data were obtained from the Scientific Registry of Transplant Recipients for each solid organ for an equidistant window before and after AC implementation. We calculated the distance between the donor and recipient hospital for each organ. We used an interrupted time series model to calculate excess travel distance after AC along with associated carbon emissions.

**Results:**

We analyzed travel distance for 226 731 deceased donor organs. There was a significant increase in total excess distance traveled: 1.5 × 10^6^ miles for lung, 3.1 × 10^6^ for heart, 2.2 × 10^6^ miles for liver, and 3.2 × 10^6^ miles for kidney. This led to increased estimated carbon emissions associated with transport ranging from: 175.7 to 193.4 kg CO2e per lung, 291.7 to 312.5 kg CO2e per heart, 114.9 to 131.7 kg CO2e per liver, and 0.2 to 5.3 kg CO2e per kidney.

**Conclusions:**

Our findings quantify an increase in total distance traveled and potential carbon emissions after AC implementation. Environmental impacts of allocation policies should be considered, especially with upcoming continuous distribution.

**Abbreviations**
AC: acuity circles
CO2e: carbon dioxide equivalent
DCD: donation after circulatory death
DSA: donor service area
HRSA: Health Resources and Services Administration
SRTR: Scientific Registry of Transplant Recipients

## 1 Introduction

Transplant allocation policies have recently changed for each solid organ, replacing donor service areas (DSAs) with 250–500 nautical mile acuity circles (AC). Depending on the organ, these changes were intended to reduce geographic variation in waiting time or urgency scores at time of transplant and minimize pre‐transplant mortality [1]. Changes were implemented on November 24, 2017 for lung, on October 18, 2018 for heart, on February 4, 2020 for liver, and for kidney and pancreas on March 15, 2021 [1, 2, 3, 4]. Since policy implementation, there have been questions raised about effectiveness, impact on mortality, cost, and travel distance [5, 6, 7].

Prior studies have consistently demonstrated that the distance traveled for all solid organ transplants has increased after the implementation of these policies [8, 9, 10]. This increase is not without potential negative consequences, such as increased ischemia time and increased complexity in arranging the organ retrieval procedures, particularly in the setting of decreasing availability of ground transport and aircraft, including the drivers and pilots [11]. Increases in distance traveled, particularly those accompanied by a change in the mode of transportation of the organ from car to plane, may also lead to increases in risk of serious accidents, exacerbation of the pilot shortage, higher costs, and increased carbon emissions and subsequent negative environmental effects [11, 12, 13, 14].

Recently, governmental agencies have called for reducing the environmental impacts of healthcare, which contributes roughly 8% of national greenhouse gas emissions each year [15]. Transplantation is an essential aspect of healthcare with high‐cost utilization, but little is known about the environmental impact of transplantation, with only one prior study quantifying the carbon costs of transplantation at a single center and with a single organ [12]. The aim of this study is to estimate the change in excess distance traveled associated with the AC change for each solid organ in the United States and quantify the resulting effects on carbon emissions.

## 2 Methods

### 2.1 Data Source and Study Population

This study used the SRTR data system, which includes data on all donors, waitlisted candidates, and transplant recipients in the United States submitted by the members of the OPTN. The Health Resources and Services Administration, US Department of Health and Human Services provides oversight to the activities of the OPTN and SRTR. contractors.

**FIGURE 1:**
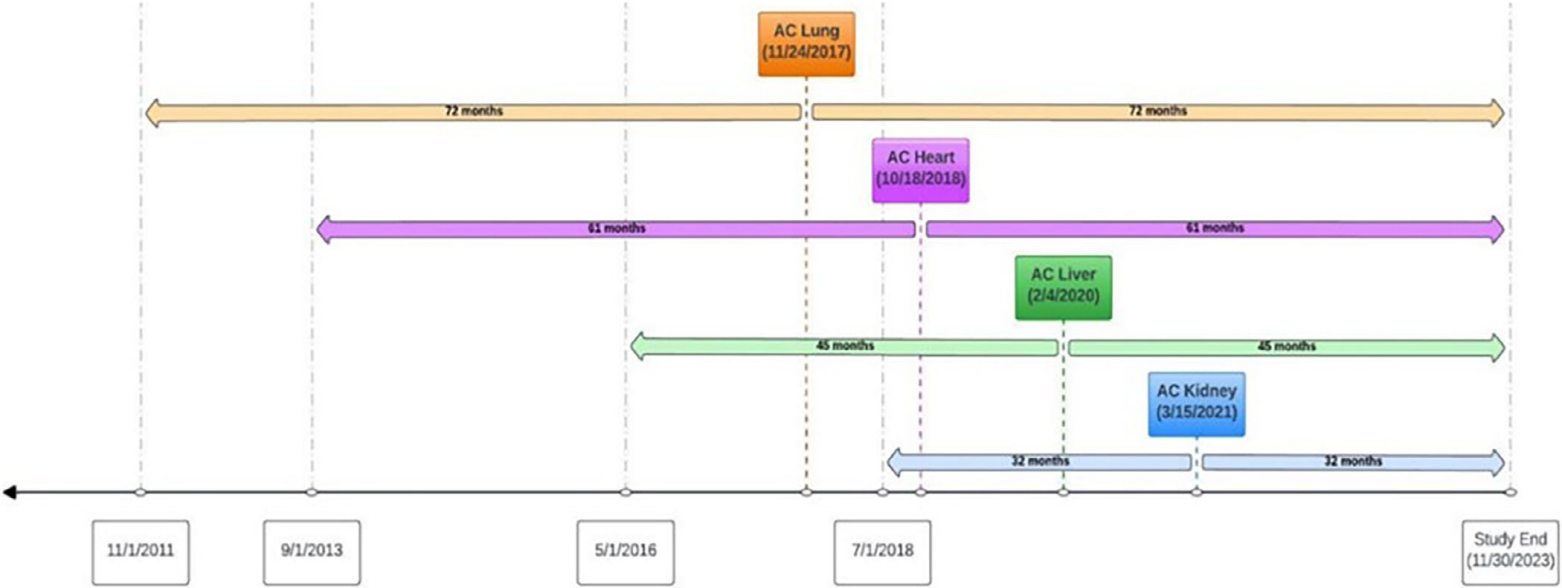
Defining organ travel observation periods for lung, heart, liver, and kidney procurements.

We defined observation periods for each included solid organ (heart, lung, liver, and kidney) centered around the implementation date of AC for each organ type, such that the number of months in the pre‐ and post‐AC cohorts were equal (Figure 1). The number of months in the post‐AC cohort was determined by the amount of time elapsed between AC implementation and the end of study data (November 30, 2023). Organs were excluded if they were from a living donor (*n* = 36 800) or if the recipient was missing a transplant center ID (*n* = 495).

### 2.2 Distance and Mode of Travel

Distance traveled by each solid organ was calculated as the distance in miles between the centroid of the donor hospital ZIP code and the receiving transplant center ZIP code using the Haversine formula. In cases where the recipient transplant center ZIP code was missing, the centroid of the center's city‐state addressed was used instead (*n* = 10).

To determine likely mode of travel (driving, charter flight, or commercial flight) for each organ, we surveyed a convenience sample of OPOs about their practices (*n* = 5; 2 in the Midwest, 2 in the West, 1 in the Southeast). Responses varied widely based on organ type and whether organs were placed on normothermic machine perfusion. Due to this variability, we decided not to set a threshold distance for travel model. Instead, we conducted our analyses with a range of assumptions. For heart, lung, and liver, we assigned travel mode based on the following assumptions: organs flown by charter if travel distance was >25 miles and driven otherwise (most liberal assumption), organs flown by charter if distance was >50 miles and driven otherwise, organs flown by charter if travel distance was >100 miles and driven otherwise, organs flown by charter if travel distance was >150 miles and driven otherwise, organs flown by charter if travel distance was >200 miles and driven otherwise (most conservative assumption). For kidney, the same assumptions were used for analysis, except that organs were assumed to travel by commercial flight instead of charter; this means that assumptions that prioritized flying over driving were more conservative.

### 2.3 Carbon Emissions

We estimated carbon emissions using data from the UK Department for Energy Security and Net Zero, reported in kilograms of carbon dioxide equivalents (kg CO2e) [16]. Driving estimates assumed that organs traveling on the ground were transported by a gasoline/petrol vehicle categorized as a “large car” [16]. For charter flights, we assumed an average of two people traveling per organ procurement, equaling an estimated 200 kg of added weight. Emissions were estimated using the UK data for short haul flights (i.e., within Europe), because the US is similar in size to Western and Central Europe combined. We considered commercial transport to not have any added carbon emissions because the organ travels with an existing flight.

### 2.4 Analyses

To determine whether travel patterns changed after AC implementation, we fit an interrupted time series model to the mean distance traveled per organ for each organ type for every month in the study period. We tested for changes in distance traveled immediately after AC implementation (changes in *intercept*) and for changes in distance trends pre‐ and post AC (changes in *slope*).

To calculate the excess distance traveled after AC implementation, we first calculated the predicted mean distance for each month based on the pre‐AC slope. This predicted mean distance was compared to the observed mean distance. We multiplied the delta between the two by the number of procurements that occurred during that month and summed this value across all months included in the post‐AC cohort.

We converted the estimated excess number of miles traveled after the implementation of AC for each organ to carbon emissions using the assumptions described above. We compared changes in mode of transport before and after the policy change for each organ and calculated excess carbon emissions based on the change in mode of transport and excess miles traveled after the policy. All analyses were conducted in R.

## 3 Results

### 3.1 Lung

There were 28 491 deceased donor lung transplants in the US between November 2011 and November 2023. Pre‐AC, the mean distance traveled per organ was 245 miles, with a non‐significant decreasing trend (*β* = −0.39, *p* for slope = 0.12). Upon AC implementation, the estimated mean distance traveled decreased by 31 miles per organ (*p* for intercept = 0.03). After this immediate decline, the month‐to‐month trend in the mean distance traveled per organ increased significantly (*β* = 3.40, *p* for slope < 0.001). The mean distance traveled per organ post‐AC was 309 miles, with a total excess distance of 1.5 million miles across all transplanted lungs (Figure 2).

FIGURE 2Interrupted time series analysis of distance traveled for transplant organ procurements before and after acuity circles implementation, by organ.

**Figure:**
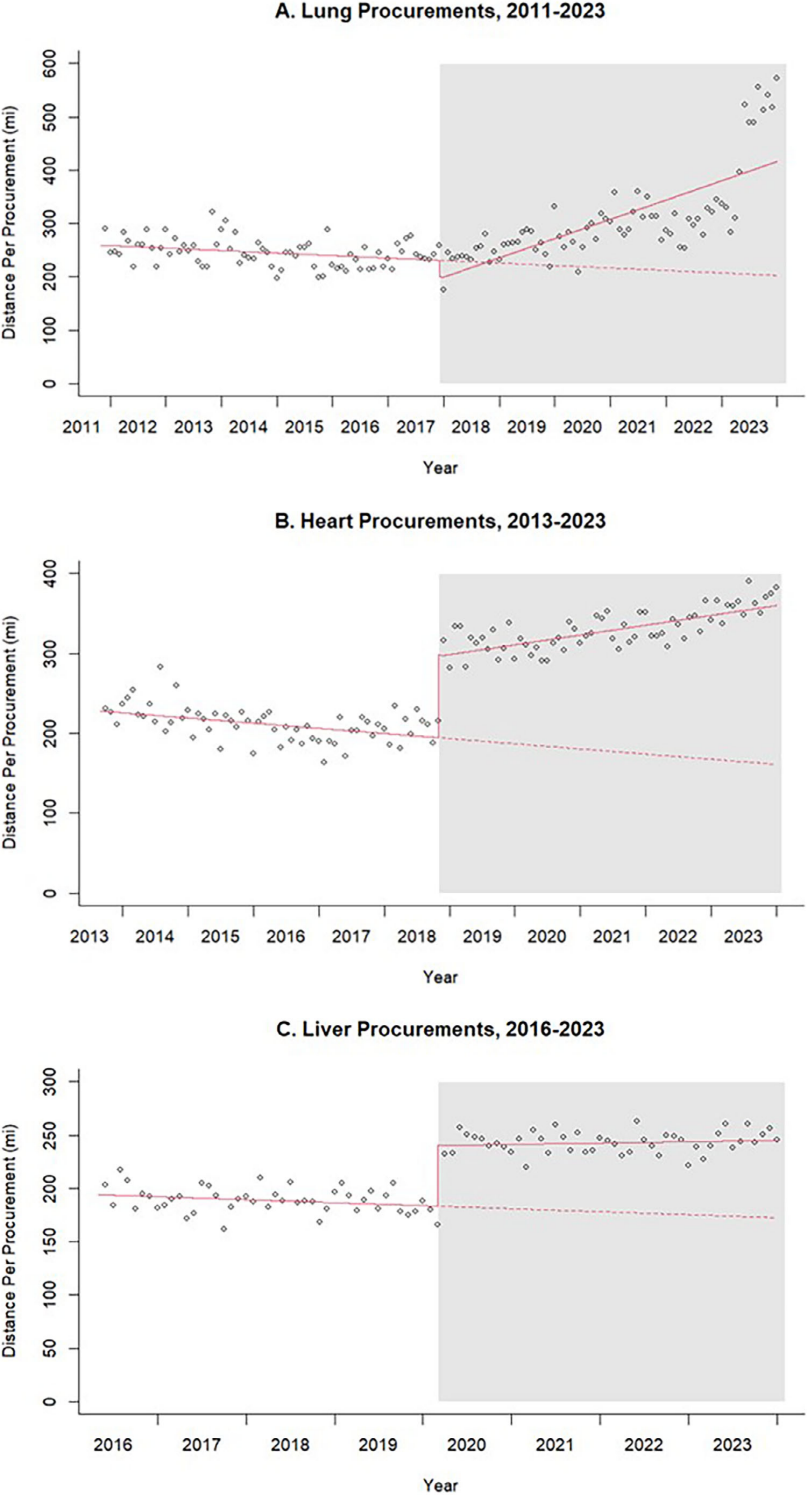

**Figure:**
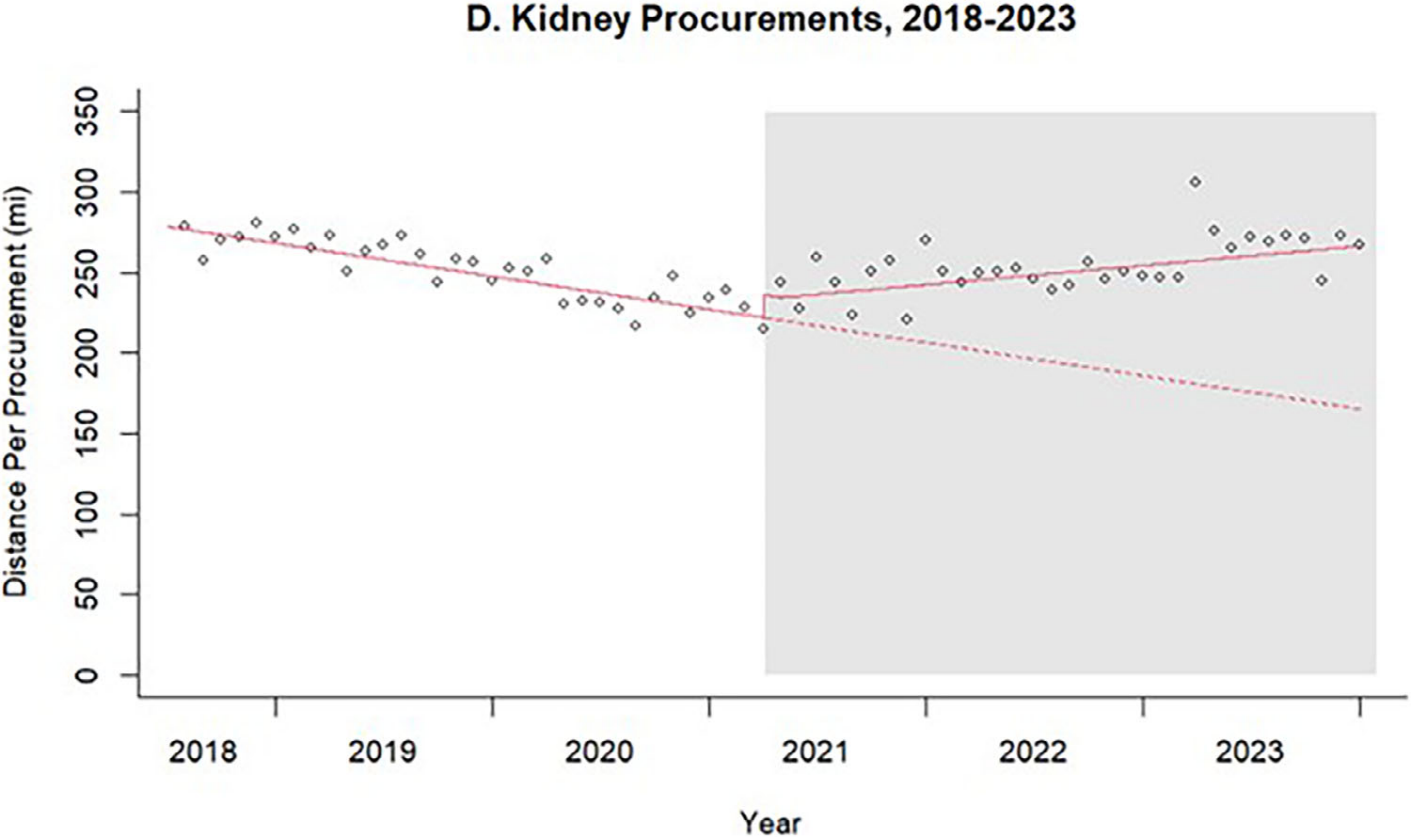

Prior to AC, 33.8% of lungs were transported less than 50 miles; after AC, the proportion of lungs traveling <50 miles declined to 18.7%. There was substantial variation across OPOs in the proportion of lungs traveling <50 miles. Prior to AC, the lowest proportion was in Region 11 (15.1%) and the highest was in Region 2 (53.8%). After AC, the lowest proportion was in Region 11 (7.2%) and the highest proportion in Region 5 (25.9%).

Per organ, excess carbon emissions associated with AC implementation ranged from 175.7 kg CO2e (lungs flown via charter for distances greater than 200 miles and driven otherwise, most conservative) to 193.4 kg CO2e (lungs were flown via charter for distances greater than 25 miles and driven otherwise, most liberal). Total excess carbon emissions ranged from 2.8 million kg CO2e (most conservative) to 3.1 million (most liberal) (Table 1). Excess emissions represented a 50% increase over pre‐AC total emissions (range of percentage increase: 50.0%–50.9%).

**TABLE 1:** Carbon emissions from organ transport attributable to excess travel distance after acuity circle implementation, by organ, in kg CO2e.

	Heart	Lung	Liver	Kidney
Fly if Dist > 25miles	6 227 941.79	3 094 439.23	4 523 546.12	9925.36
Fly if Dist > 50miles	6 208 442.85	3 079 895.36	4 488 056.06	24 104.43
Fly if Dist > 100miles	6 125 572.32	3 026 567.85	4 356 742.87	79 402.84
Fly if Dist > 150miles	5 998 829.17	2 936 880.67	4 175 743.60	161 641.50
Fly if Dist > 200miles	5 813 589.17	2 810 833.82	3 945 058.26	282 163.67

### 3.2 Heart

There were 35 404 deceased donor heart transplants between September 2013 and November 2023. Pre‐AC, the mean distance traveled per organ was 212 miles, with a significant month‐to‐month decreasing trend (*β* = −0.54, *p* for slope < 0.001). After AC implementation, the estimated mean distance traveled per procurement increased by 104 miles per organ (*p* for intercept < 0.001). The month‐to‐month trend also changed to an increasing slope (*β* = 1.56, *p* for slope < 0.001). Post‐AC, the mean distance traveled per organ was 330 miles, with a total excess of 3.1 million miles across all heart transplants.

Prior to AC, 37% of hearts were transported < 50 miles; after AC, this proportion dropped to 15.5%. OPO Region 11 had the lowest proportion of hearts traveling < 50 miles prior to AC, while OPO Region 9 had the highest (56.8%). After AC, OPO Region 10 had the lowest proportion of hearts traveling < 50 miles (6.8%); Region 9 continued to have the highest (32.0%).

Per organ, excess carbon emissions associated with AC implementation ranged from 291.7 kg CO2e (most conservative) to 312.5 kg CO2e (if all hearts were flown by charter, most liberal). Total potential excess carbon emissions from heart transport after AC ranged from 5.8 million kg CO2e (most conservative) to 6.2 million kg CO2e (most liberal) (Table 1). The estimated percentage increase in carbon emissions from total pre‐AC emissions ranged from 94.5% (if all flown by charter) to 100.2% (if all traveling > 200 miles were flown by charter and the rest driven).

### 3.3 Liver

There were 64 119 deceased donor liver transplants between May 2016 and November 2023. Pre‐AC, the mean distance traveled per organ was 189 miles, with a non‐significantly decreasing trend (*β* = −0.24, *p* = 0.06). After AC implementation, the distance traveled per procurement abruptly increased by 57 miles per organ (*p* for intercept < 0.001), with no significant change in slope (*β* = 0.34, *p* for slope = 0.05). The mean distance traveled per organ post‐AC was 243 miles, with a total excess of 2.2 million miles across all liver transplants.

Prior to AC, 52.6% of livers were transported < 100 miles; after AC, this proportion decreased to 35.9%. The OPO region with the lowest proportion of livers traveling < 100 miles was Region 11, both pre‐AC (42.5%) and post‐AC (20.3%). The OPO region with the highest proportion of lowest traveling < 100 miles was Region 1, both pre‐AC (75.1%) and post‐AC (49.0%).

Per organ, excess carbon emissions after AC implementation ranged from 114.9 kg CO2E per liver (most conservative) to 131.7 kg CO2E per liver (most liberal). The total range of excess carbon emissions from liver transport after AC implementation was calculated as 3.9 million kg CO2e (most conservative) to 4.5 million kg CO2e (all organs flown by charter) (Table 1). This excess carbon emission represented a percentage increase from total pre‐AC emissions of ∼40% (range: 39.7%–41.0%).

### 3.4 Kidney

There were 98 717 deceased donor kidney transplants in the US between July 2018 and November 2023. Pre‐AC, the mean distance traveled per organ was 251 miles with a significant decreasing trend (*β* = −1.72, *p* for slope < 0.001). Immediately after AC, the estimated mean distance traveled per procurement increased by 14 miles per organ (*p* for intercept = 0.02). The month‐to‐month trend also changed to an increasing slope (*β* = 2.71, *p* for slope < 0.001). Post‐AC, the mean distance traveled per organ was 254 miles with a total excess distance of 3.2 million miles across all kidney transplants.

The proportion of kidneys that traveled <200 miles was 70.7% prior to AC and 64.4% after AC. OPO Region 6 had the lowest proportion of kidneys that traveled <200 miles both pre‐AC (52.3%) and post‐AC (54.1%). Prior to AC, OPO Region 9 had the highest proportion of kidneys that traveled <200 miles (85.3%); after AC, OPO Region 1 had the highest proportion (78.1%).

Per organ, excess carbon emissions after AC implementation ranged from 0.2 kg CO2e (if all kidneys transported >25 miles were flown via pre‐existing commercial transport and driven otherwise) to 5.3 kg CO2e per kidney (if all kidneys transported >200 miles were flown via pre‐existing commercial transport and driven otherwise).Total excess carbon emissions attributed to kidney transport after AC ranged from 9.925 kg CO2e (most conservative) to 282 163 kg CO2e (most liberal) (Table 1). The estimated percentage increase from pre‐AC emissions ranged from 19.7% (if all kidneys traveling > 25 miles were flown) to 36.2% (if all kidneys traveling > 200 miles were flown).

### 3.5 All Organs

After AC implementation, carbon emissions attributable to the excess distance traveled ranged from 12.6 million kg CO2e (most conservative assumption set, a 56.6% increase) to 14.1 million kg CO2e (most liberal assumption set, a 52.2% increase). This total corresponds to a range of 101.5–114.0 excess kg CO2e per organ.

## 4 Discussion

This study quantified increases in distance traveled by donor organs after AC policy implementation for lung, heart, liver, and kidney transplantation in the United States. We found that there was a significant increase in total excess distance traveled for each organ in the time period after AC implementation: 1.5 million miles for lung transplants, 3.1 million miles for heart transplants, 2.2 million miles for liver transplants, and 3.2 million miles for kidney transplants. In the most conservative scenario, carbon emissions attributable to this excess travel totaled 12.6 million kg CO2e, a 56% overall increase from pre‐AC levels. In the most liberal scenario, excess carbon emissions from increased travel distance exceeded 14 million kg CO2e. Our findings highlight the importance of considering environmental impacts of proposed organ allocation policies and identifying strategies to mitigate increased carbon emissions for upcoming continuous distribution.

Our findings are consistent with prior studies that demonstrated increased travel for organs after AC implementation [8, 9, 10]. In contrast to other studies, we focused on *excess* travel distance by using interrupted time series models to predict what travel distance would have been in the absence of AC implementation. This allowed us to separate the effects of AC implementation more precisely from secular trends. Increased organ travel distance can have several negative effects, both clinically and structurally. Increased travel distance can lead to increased cold ischemic time, which becomes especially relevant for trips over 3 h and may lead to worse transplant outcomes [10, 17]. Cost also increases with increased distance traveled. One analysis of the cost of liver transplant after ACs found increased overall fees associated with transplant, driven mostly by increased surgeon, import, and charter fees [13]. Policies to limit travel distances for organs may result in reduced costs to the overall health system. This is especially relevant for lungs, hearts, and kidneys, where the number of miles traveled monthly is increasing significantly, suggesting continued increases in the future. These month‐to‐month increases may reflect center responses to increasingly complex organ offer environment or a growing comfort with organs transported long distances, potentially due to increased use of mitigating technology like machine perfusion.

ACs implementation was accompanied by several other secular trends in transplantation, including the COVID‐19 pandemic, an increase in donation after circulatory death (DCD) procurement, and the rise of normothermic perfusion technology. In particular, the latter may impact our findings, as travel considerations change based on whether perfusion was used and what type (i.e., back‐to‐base, third party vendors). For some types of perfusions, organs will nearly always be driven. Current data collection practices do not capture back‐to‐base perfusion or travel model, forcing our reliance on a series of assumptions. However, even in our most conservative estimates, we identified a substantial increase in potential carbon emissions associated with excess travel distance after AC implementation.

Increased carbon emissions have adverse environmental and health effects [18, 19]. The increase in carbon emissions observed in this study were driven by travel distance and changes in mode of transport from ground to charter flight [20]. We quantified potential carbon emission impacts from our calculated excess distance. Using the US Environmental Protection Agency Greenhouse Gas Equivalence calculator, the total increase in kg CO2e after the AC policy change calculated by our study is equivalent to any of the following ranges based on most and least conservative estimates: 1236 to 6834 gasoline‐powered passenger vehicles driven for 1 year, 13 496 766 to 74 614 195 miles driven by average gasoline‐powered passenger vehicles, 596 377 to 3 296 951 gallons of gasoline consumed, 1104 to 6106 homes’ electricity use for 1 year, or 5 887 319 to 32 546 876 pounds of coal burned [20]. On a per‐organ basis, excess carbon emissions after AC implementation ranged anywhere from the equivalent of 0.2 (kidneys predominantly flown by commercial air) to the equivalent of 350 pounds of coal burned (hearts predominantly flown by charter). These findings demonstrate that changes in organ allocation policy can translate into measurable increases in carbon emissions, reflecting an often‐overlooked environmental consequence of system‐level policy shifts.

Practices vary significantly among organs. Kidneys are often transported by commercial aircraft, resulting in our findings of the relatively lower environmental impact of kidney transport compared to other organs. Because we do not have data on travel mode, we provide a range of scenarios varying from most conservative (non‐kidney organs flown by charter if travel distance was >200 miles) to most liberal (non‐kidney organs flown by charter if travel distance was >25 miles). However, even our most conservative scenarios may be underestimates, as we analyzed the distance that the organ traveled after procurement. This distance does not include any additional travel from the team to the donor hospital, transportation to a donor recovery facility prior to allocation, or courier costs associated with commercial flights. Further, we were unable to include “dry‐runs”, or non‐utilized organs. Therefore, our estimates likely represent a lower bound on the true logistical and financial burden associated with organ transportation.

Reducing unnecessary air transport has been identified as a point of maximal leverage for reducing carbon emissions [21, 22]. Although the exact percentage of air versus ground transport for organ procurements varies by center, one study found that the number of fly‐outs and dry‐runs increased for liver transplant after the AC policy change [23]. In our study, we found that after AC implementation, the proportion of donor organs that were likely to travel by charter flight versus ground transportation based on distance increased significantly for all organs except kidneys. The increased distance traveled and subsequent increase in air transport also requires increased coordination among teams to organize flights. This can increase costs with added complexity and allow more room for error, as well as potentially decreased safety [24, 25]. Notably, our estimates showed that the environmental impact of commercial transport was much lower than dedicated charter flights. In 2025, the Federal Aviation Administration (FAA) Organ Transport Working Group a report to facilitate safer, standardized, and more effective organ transport by commercial flight, which may promote additional renal and non‐renal transport and reduce the need for charter flight usage [2].

Further changes to the allocation policy are now on the horizon. Continuous distribution was implemented for lungs on March 9, 2023 and is currently under development for other organs [26]. We anticipate that travel distance and associated carbon emissions will rise under continuous distribution. Signals of this increase can be seen in the past year for lung, although data are currently too limited to estimate the effects with precision. Organ allocation policy is designed to prioritize minimizing the number of deaths of the waitlist, which is the highest goal of the transplant system. However, recent debates have suggested that other goals may also need to be considered when evaluating allocation policies, including efficiency and logistical burden. Our findings demonstrate that allocation policy has substantial influence on the environmental impacts of the transplantation system, which may be another important element of policy evaluation. As allocation policies continue to move toward continuous distribution, incorporating considerations of efficiency, logistical feasibility, and environmental impact alongside survival may promote a more sustainable and resilient transplant system.

Secular trends may have impacted our analyses. The time course of AC policy implementation was different for each organ, and the external factors of changing transplant practices and the effects of the COVID‐19 pandemic in 2020 may have affected procurement practices [27]. In addition, machine perfusion use has expanded dramatically in recent years, increasing from 40% of kidneys in January 2016% to 72% in August 2025 and from 1% to 34% of livers and 1% to 20% of hearts in the same time period [3]. It is unclear how the use of machine perfusion or the type of perfusion (on site vs. back to base) may impact emissions as organs are still managed with similar time constraints and transportation modes, with the possible exception of pumped kidneys which may be more inclined to opt for ground transportation rather than air. In our analyses, this scenario led to increased carbon emissions associated with kidney transport, although emissions were still lower for kidney than for any other organ.

This analysis is subject to other limitations. We do not have data on mode of transport and relied on assumptions based on travel distance. Although we conducted a survey of a convenience sample of OPOs, these OPOs did not represent all regions and responses may not be generalizable across the country. We conducted several sensitivity analyses to evaluate the robustness of our findings to these assumptions. Additional assumptions (i.e., the use of “large vehicles” instead of electric vehicles) may also influence calculated emissions.

## 5 Conclusion

This study demonstrates an increase in the excess distance traveled for each organ after the implementation of AC policy, accompanied by a substantial increase in carbon emissions from organ transport. Continuous distribution will likely further increase travel distances and resulting environmental impact. Potential environmental impacts should be considered during allocation policy development and evaluation.

## Conflicts of Interest

The authors declare no conflicts of interest.

## Data Availability

The data that support the findings of this study are available from the Scientific Registry of Transplant Recipients. Restrictions apply to the availability of these data, which were used under license for this study. Data are available from https://www.srtr.org/requesting‐srtr‐data/about‐srtr‐standard‐analysis‐files/ with the permission of the Scientific Registry of Transplant Recipients.

## References

[1] System Notice; Liver and Intestinal Organ Distribution Based on Acuity Circles Implemented Feb. 4. Published online February 4, 2020. Accessed June 9, 2022, https://unos.org/news/system‐implementation‐notice‐liver‐and‐intestinal‐organ‐distribution‐based‐on‐acuity‐circles‐implemented‐feb‐4/.

[2] Y. Becker . Services USDoHaH. Published online November 21, 2017. Accessed March 27, 2024, https://optn.transplant.hrsa.gov/media/2397/hrsa_letter_to_optn_20171121.pdf.

[3] OPTN Public Comment Document. Proposal to Modify the Adult Heart Allocation System. Published online August 2016. Accessed March 15, 2024, https://optn.transplant.hrsa.gov/media/1921/thoracic_adult_heart_allocation_modification_20160815.pdf.

[4] New Kidney, Pancreas Allocation Policies in Effect. Published online March 15, 2021. Accessed March 27, 2024, https://optn.transplant.hrsa.gov/news/new‐kidney‐pancreas‐allocation‐policies‐in‐effect/.

[5] V. Puri , R. R. Hachem , C. C. Frye , et al., “Unintended Consequences of Changes to Lung Allocation Policy,” American Journal of Transplantation 19, no. 8 (2019): 2164–2167, 10.1111/ajt.15307. 30758137 PMC6658330

[6] S. R. Kumar , D. Chyou , and D. Goldberg , “Effect of Acuity Circles Allocation Policy on Local Use of Donation After Circulatory Death Donor Livers,” Liver Transplantation 28, no. 6 (2022): 1103–1107, 10.1002/lt.26402. 35000270

[7] R. R. Goff , K. Uccellini , K. Lindblad , et al., “A Change of Heart: Preliminary Results of the US 2018 Adult Heart Allocation Revision,” American Journal of Transplantation 20, no. 10 (2020): 2781–2790, 10.1111/ajt.16010. 32406597

[8] UNOS Research Department. Committee OT. Monitoring of the Lung Allocation Change, 6‐Month Report Removal of DSA as a Unit of Allocation. Published online June 29, 2018. Accessed April 1, 2024, https://optn.transplant.hrsa.gov/media/2517/20180621_thoracic_committee_report_lung.pdf.

[9] K. H. Sheetz and S. A. Waits , “Outcome of a Change in Allocation of Livers for Transplant in the United States,” JAMA Surgery 156, no. 5 (2021): 496, 10.1001/jamasurg.2021.0137. 33729439 PMC7970393

[10] Eliminate Use of DSA and Region From Kidney Allocation. Two Year Post‐Implementation Monitoring Report. Published online June 22, 2023. Accessed April 1, 2024, https://optn.transplant.hrsa.gov/media/4mhfm3oq/eliminate_use_of_dsa_and_region_from_kidney_allocation_two_year_post_implementation_monitoring_report_2yr.pdf.

[11] When Minutes Matter. Published online February 15, 2023. Accessed March 27, 2024, https://unos.org/news/insights/when‐minutes‐matter‐organ‐transportation/.

[12] A. E. Wall , T. Borries , V. Reddy , S. K. Asrani , G. Testa , and J. Trotter , “The Carbon Footprint of Organ Acquisition in the United States,” American Journal of Transplantation 22, no. 12 (2022): 3184–3185, 10.1111/ajt.17196. 36088644

[13] A. E. Wall , G. B. Da , and S. K. Asrani , “Cost Analysis of Liver Acquisition Fees Before and After Acuity Circle Policy Implementation,” JAMA Surgery 156, no. 11 (2021): 1051, 10.1001/jamasurg.2021.4406. 34495291 PMC8427489

[14] A. D. Schenk , W. K. Washburn , A. B. Adams , and R. J. Lynch , “A Survey of Current Procurement Travel Practices, Accident Frequency, and Perceptions of Safety,” Transplant Direct 5, no. 10 (2019): e494, 10.1097/TXD.0000000000000942. 31723589 PMC6791602

[15] Delivering a net zero NHS. Published online July 4, 2022. Accessed April 1, 2024, https://www.england.nhs.uk/greenernhs/wp‐content/uploads/sites/51/2022/07/B1728‐delivering‐a‐net‐zero‐nhs‐july‐2022.pdf.

[16] CO2 Emissions of Transport Modes (UK Government) “Transport Emissions Per Kilometer Travelled.” Published online September 20, 2022. Accessed April 25, 2024, https://www.gov.uk/government/publications/greenhouse‐gas‐reporting‐conversion‐factors‐2022.

[17] D. A. DuBay , P. A. MacLennan , R. D. Reed , et al., “The Impact of Proposed Changes in Liver Allocation Policy on Cold Ischemia Times and Organ Transportation Costs,” American Journal of Transplantation 15, no. 2 (2015): 541–546, 10.1111/ajt.12981. 25612501 PMC4429785

[18] N. L. Mills , K. Donaldson , P. W. Hadoke , et al., “Adverse Cardiovascular Effects of Air Pollution,” Nature Clinical Practice Cardiovascular Medicine 6, no. 1 (2009): 36–44, 10.1038/ncpcardio1399. 19029991

[19] P. R. Hunter , “Climate Change and Waterborne and Vector‐Borne Disease: CLIMATE CHANGE and DISEASE,” Journal of Applied Microbiology 94 (2003): 37–46, 10.1046/j.1365-2672.94.s1.5.x. 12675935

[20] United States Environmental Protection Agency . Greenhouse Gas Equivalencies Calculator (March 2022), Accessed March 25, 2024, https://www.epa.gov/energy/greenhouse‐gas‐equivalencies‐calculator.

[21] C. Ewbank , B. Stewart , B. Bruns , et al., “Introduction of the Surgical Providers Assessment and Response to Climate Change (SPARC2) Tool: One Small Step Toward Reducing the Carbon Footprint of Surgical Care,” Annals of Surgery 273, no. 4 (2021): e135–e137, 10.1097/SLA.0000000000004367. 33214422

[22] C. Ewbank , B. Stewart , B. Bruns , et al., “The Development of a Surgical Care and Climate Change Matrix: A Tool to Assist With Prioritization and Implementation Strategies,” Annals of Surgery 273, no. 2 (2021): e50–e51, 10.1097/SLA.0000000000003980. 32404663

[23] O. Ahmed , M. B. Doyle , M. S. Abouljoud , et al., “Liver Transplant Costs and Activity After United Network for Organ Sharing Allocation Policy Changes,” JAMA Surgery 159, no. 8 (2024): 939–947, 10.1001/jamasurg.2024.1208. 38809546 PMC11137658

[24] NHTSA Estimates Traffic Fatalities Continued to Decline in the First Half of 2023. Published online September 28, 2023. Accessed April 17, 2024, https://www.nhtsa.gov/press‐releases/2023‐Q2‐traffic‐fatality‐estimates.

[25] M. J. Englesbe and R. M. Merion , “The Riskiest Job in Medicine: Transplant Surgeons and Organ Procurement Travel,” American Journal of Transplantation 9, no. 10 (2009): 2406–2415, 10.1111/j.1600-6143.2009.02774.x. 19663887

[26] S. Weiss and C. Hawkins , “Lung Continuous Distribution One Year Monitoring Report,” (2024), Accessed September 7, 2024, https://optn.transplant.hrsa.gov/media/srino34s/data_report_lung_cd_1year_20240509.pdf.

[27] A. Loupy , O. Aubert , P. P. Reese , O. Bastien , F. Bayer , and C. Jacquelinet , “Organ Procurement and Transplantation During the COVID‐19 Pandemic,” Lancet 395, no. 10237 (2020): e95–e96, 10.1016/S0140-6736(20)31040-0. 32407668 PMC7213957

